# Insights Into Thiamine Supplementation in Patients With Septic Shock

**DOI:** 10.3389/fmed.2021.805199

**Published:** 2022-01-28

**Authors:** Nara Aline Costa, Amanda Gomes Pereira, Clara Sandra Araujo Sugizaki, Nayane Maria Vieira, Leonardo Rufino Garcia, Sérgio Alberto Rupp de Paiva, Leonardo Antonio Mamede Zornoff, Paula Schmidt Azevedo, Bertha Furlan Polegato, Marcos Ferreira Minicucci

**Affiliations:** ^1^Faculty of Nutrition, Universidade Federal de Goiás (UFG), Goiânia, Brazil; ^2^Department of Internal Medicine, Botucatu Medical School, São Paulo State University (UNESP), Botucatu, Brazil

**Keywords:** thiamine supplementation, thiamine deficiency, septic shock, vitamin B1, mitochondrial dysfunction

## Abstract

Septic shock is associated with unacceptably high mortality rates, mainly in developing countries. New adjunctive therapies have been explored to reduce global mortality related to sepsis. Considering that metabolic changes, mitochondrial dysfunction and increased oxidative stress are specific disorders within the path of septic shock, several micronutrients that could act in cellular homeostasis have been studied in recent decades. Thiamine, also known as vitamin B1, plays critical roles in several biological processes, including the metabolism of glucose, synthesis of nucleic acids and reduction of oxidative stress. Thiamine deficiency could affect up to 70% of critically ill patients, and thiamine supplementation appears to increase lactate clearance and decrease the vasopressor dose. However, there is no evident improvement in the survival of septic patients. Other micronutrients such as vitamin C and D, selenium and zinc have been tested in the same context but have not been shown to improve the outcomes of these patients. Some problems related to the neutrality of these clinical trials are the study design, doses, route, timing, length of intervention and the choice of endpoints. Recently, the concept that multi-micronutrient administration may be better than single-micronutrient administration has gained strength. In general, clinical trials consider the administration of a single micronutrient as a drug. However, the antioxidant defense is a complex system of endogenous agents in which micronutrients act as cofactors, and the physiological interactions between micronutrients are little discussed. In this context, the association of thiamine, vitamin C and corticoids was tested as an adjunctive therapy in septic shock resulting in a significant decrease in mortality. However, after these initial results, no other study conducted with this combination could reproduce those benefits. In addition, the use of low-dose corticosteroids is recommended in patients with septic shock who do not respond to vasopressors, which can affect the action of thiamine. Therefore, given the excellent safety profile, good biologic rationale and promising clinical studies, this review aims to discuss the mechanisms behind and the evidence for single or combined thiamine supplementation improving the prognosis of patients with septic shock.

## Introduction

Septic shock is a subset of sepsis characterized by profound hemodynamic alterations associated with organ dysfunction and is one of the most common causes of admission to intensive care units (ICUs) (1). Despite advances in management, rates of sepsis are still rising worldwide, and it is associated with high morbidity, disability and mortality (2).

**Figure 1:**
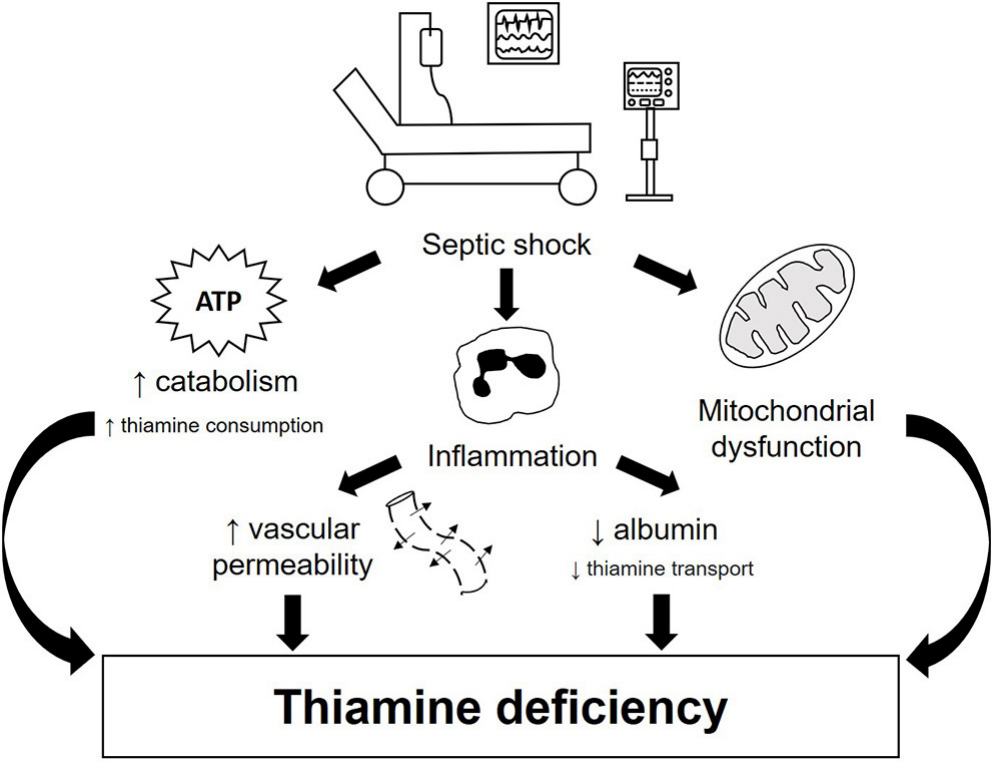
Pathophysiology of thiamine deficiency in septic shock patients. Thiamine deficiency in septic shock occurs both due to increased need and impaired transport. This condition can be triggered by three main mechanisms: hypercatabolism, exacerbated pro-inflammatory response and mitochondrial dysfunction. During septic shock, there is an increase in the metabolic demand for energy, resulting in increased glycogenolysis, inhibition of glycogenesis and increased production of glucose via gluconeogenesis, considerably increasing the need for thiamine for glucose metabolism. Inflammation, on the other hand, promotes increased vascular permeability and reduced albumin production, impairing thiamine transport. In addition, tissue hypoxia present in septic shock is one of the main triggers of mitochondrial dysfunction, which contributes to an imbalance in glucose homeostasis, including lower availability of ATP and increased serum lactate concentration. All these mechanisms together contribute to the development and/or worsening of thiamine deficiency in patients with septic shock.

In critical illness, and most notably in sepsis, the metabolic response to trauma, although necessary, can usually overwhelm the body's metabolism, leading to a wide range of clinical consequences. This response implies significant changes in intermediary metabolism, including increased glycogenolysis, inhibition of glycogenesis and increased lipolysis, producing glucose via gluconeogenesis of lactate, glycerol and amino acids (3). In this scenario, some vitamins and minerals are essential for energy metabolism and mitochondrial function; among these, thiamine deserves to be highlighted (4, 5). Currently, the role of thiamine in sepsis treatment has become of particular interest. Thiamine deficiency might be involved in the pathophysiology of septic shock because high serum lactate concentrations, metabolic acidosis and hypotension can occur in both conditions (6) (Figure 1).

The evidence of thiamine deficiency in critically ill patients was discovered in the 1980s in patients who developed classical clinical manifestations, such as cardiac disorders and neuropsychiatric syndromes, after ICU admission (7–9). Afterwards, thiamine body status levels were first assessed in critically ill patients by Cruickshank et al. (10), who reported deficiency in 20% of adult patients upon admission to the ICU. Also, higher thiamine body status levels were associated with lower mortality. Despite important limitations, this previous investigation brought to light a considerable concern that thiamine deficiency might be related to poorer outcomes or even could be a potentially life-threatening condition in critically ill patients.

Indeed, thiamine deficiency is relatively prevalent in septic shock patients, with rates as high as 71.3% (11). In addition, it is essential to note that in sepsis, there are some severe metabolic derangements, so improvement in organ failure is essential. In this way, antioxidant–enforcement and mitochondrial stress attenuation are specific targets for the rationale of thiamine treatment during septic shock states (12). However, the results of studies assessing thiamine supplementation effects on outcomes remain inconsistent (11, 13–15).

Thus, understanding thiamine's multiple functions in several biological processes by examining past and ongoing studies will lead to the further definition of potential targets for septic shock treatment. This review aims to discuss the mechanisms and the evidence for single or combined thiamine supplementation on the prognosis of patients with septic shock.

## Thiamine: Functions and Mechanisms of Action

Thiamine or vitamin B1 is a water-soluble and thermosensitive vitamin that is not produced by our body and is indispensable in the human diet. It is considered an essential component of cell metabolism and is mainly involved in glucose metabolism (16).

In 9th-century appeared in Japan first descriptions of states associated with thiamine deficiency in the form of beriberi (17). Since then, knowledge about thiamine has increased along with the gradual recognition of diseases associated with its deficiency, which are divided into two classical clinical forms: encephalopathy (peripheral neuropathy and Wernicke-Korsakoff) and beriberi, which can be classified into dry beriberi (muscle weakness and anorexia), wet beriberi (high-output heart failure) and Shoshin beriberi (beriberi associated with shock) (18–20).

Thiamine is naturally present in several foods, such as meat (especially lean cuts of pork), cereals, yeasts, grains, fruits and other products of plant origin (21). Its biochemical structure consists of a thiazole ring and a pyrimidine group that together make up the sulfur-containing structure of two rings joined by a methylene group (16). Both fractions, pyrimidine and thiazole rings are necessary for thiamine biological activity (22). Its molecule can suffer the action of thiaminases, which are enzymes that cleave the thiamine in the methylene bridge, inactivating it. Thiaminases are produced by bacteria present in the small bowel and colon and can also be found in some raw foods such as fish and shellfish (22). Thus, frequent intake of these foods heightens the risk of thiamine deficiency.

Six thiamine compounds are known in metabolism: free thiamine; thiamine monophosphate (TMP); thiamine diphosphate, also referred to as thiamine pyrophosphate (TPP); adenosine thiamine diphosphate (ATDP); thiamine triphosphate and adenosine thiamine triphosphate (17). The most important biological active form is TPP, and it accounts for 80–90% of total body thiamine content (17, 23). Approximately 90% of the total thiamine in the blood is found in erythrocytes (75%) and leukocytes (15%) in the TPP form (24). About 5–15% of the total vitamin B1 is in the form of free thiamine, which is bound to albumin in the blood or as TMP form. The remaining forms of vitamin B1 account for only 1% of the total thiamine in humans (17, 24).

There are no significant stores of thiamine in any human tissue (21), and its concentrations are highest in the skeletal muscles, heart, kidney, liver and brain respectively (24, 25). Due to its short half-life of 9.5–18.5 days (24), necessary role in multiple metabolic processes and increased requirements in some pathological states, appropriate dietary intake is crucial for avoiding deficiency states (26, 27).

The Recommended Daily Allowance (RDA) of thiamine for healthy adults is 1.1–1.2 mg/day (21). However, in individuals at risk or with established thiamine deficiency (TD) there is no consensus on the appropriate dose, frequency, or duration of supplementation. In critically ill patients, prophylaxis or treatment for TD typically consists of parenteral administration of thiamine (25, 26). The intravenous route is most frequently used due to rapidly achieving high plasma concentrations, flexible rate of dosing, and better site tolerance as compared to intramuscular injection (28). Also, thiamine replacement by oral or enteral route is possible in situations of non-emergent deficiency, though it is important to note that the gastrointestinal microenvironment is often perturbed during sepsis, resulting in gut dysfunction and nutrient malabsorption (24, 29).

Although thiamine is considered to have a very good safety profile, the tolerable upper intake level (UL) is not established (30). Studies have shown that parenteral doses >500 mg have occasionally led to anaphylaxis and minimal adverse effects such as nausea, anorexia, lethargy, mild ataxia, and a diminution of gut tone (26, 28, 31).

Considering the availability and half-life, the most recommended methods to detect thiamine deficiency are the assessment of transketolase activity (which uses TPP as a cofactor) and the measurement of ATDP, both of which conducted in erythrocytes (24, 32). However, the lack of availability and high costs of these methods limit their use in clinical practice (33).

**Figure 2:**
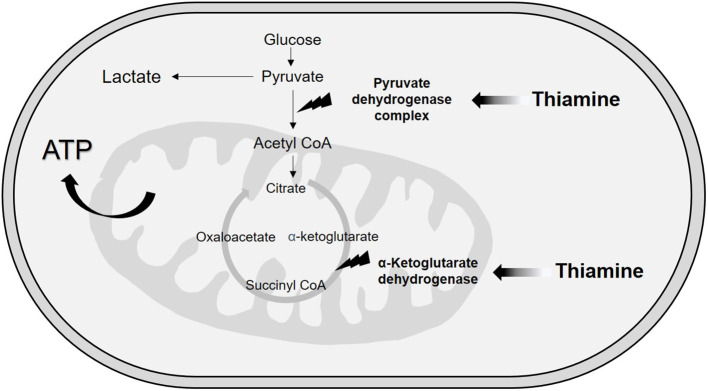
Metabolic function of thiamine.

Although vitamin B1 is involved in several related and simultaneous biological processes, we can divide the functions of thiamine into metabolic or enzymatic and structural or nonenzymatic roles (34, 35). Concerning metabolic functions, thiamine plays an essential role in energy transformation because TPP is a cofactor of two enzymes related to the extraction of energy from carbohydrate sources (16, 24, 34). These mitochondrial enzymes are involved in decarboxylation reactions and dehydrogenation reactions (24). The first enzyme is the multienzyme complex pyruvate dehydrogenase (PDH), which is made up of TPP-dependent pyruvate decarboxylase, a lipoic acid-dependent dihydrolipoyl transacetylase and a flavin adenine dinucleotide (riboflavin)-dependent dihydrolipoyl dehydrogenase (24, 36). In the related PDH reactions, TPP receives electrons in the redox processes (16), resulting in the conversion of pyruvate to acetyl-CoA, which then takes place in the Krebs cycle (37). At this point, we can infer that in states of thiamine deficiency, pyruvate access to the mitochondria is impaired with cytosolic conversion to lactate via lactate dehydrogenase and further lactic acidosis (Figure 2).

TPP is also a cofactor for α-ketoglutarate dehydrogenase, a mitochondrial enzyme responsible for converting α-ketoglutarate to succinyl-CoA in the Krebs cycle (24) through a decarboxylation reaction (16). This function highlights crucial redox mechanisms taking place inside the mitochondria, which is responsible for over 95% of adenosine 5-triphosphate generation and, consequently, oxygen reactive species production (12, 16). Mitochondrial dysfunction is well known in sepsis (12), and potential targets for thiamine supplementation might be located inside this organelle.

In turn, TPP is also useful in non-oxidative carbohydrate metabolism. The cytosolic enzyme transketolase needs TPP as a cofactor (24). This reaction is found within the pentose phosphate pathway, where sugars are interconverted and are essential for pentoses generation to nucleic acids synthesis and nicotinamide adenine dinucleotide phosphate (NADPH) for the production of fatty acids, maintenance of myelin sheaths, nerve membrane function and signal transmission (16, 24). At this point, both functions of thiamine are interconnected. NADPH, in turn, is involved in glutathione cycling, an important antioxidant pathway and potential target of thiamine as a metabolic resuscitator in patients with septic shock (38). It is also important to emphasize that thiamine requires magnesium as a cofactor for conversion into its TPP active form, playing a key role in aerobic metabolism as well. Magnesium absence, therefore, may result in altered metabolism of glucose and increased lactate production (39). Hence, in clinical practice, the correction of concomitant magnesium deficiency is essential to thiamine utilization, although it is often overlooked (40, 41).

Regarding the nonenzymatic functions of vitamin B1, we can highlight its relevance in the nervous system and its interface with the immune system. In the nervous system, thiamine is related to the transmission of nerve impulses. Playing important roles in sodium permeability, TPP is also involved in maintaining negative charge on the inner surface of the cell membranes and facilitates neurotransmission by acting on the release of some neurotransmitters, notably acetylcholine (24). It was demonstrated that thiamine deficiency provoked a significant decrease in the voltage-dependent K+ membrane conductance of cerebellar neurons, mainly by suppressing an A-type K+ channel, which leads to important neuronal alterations (34) and a further reduction of nervous conduction velocity. In addition, TPP's role in fatty acid and NADPH synthesis is related to adequate neurologic functions (24).

In the immune system, thiamine has several functions in the regulation and activation of immune cells and proteins (16). The balance between glycolysis and Krebs cycle activities, where vitamin B1 is an important cofactor, is considered a determinant in controlling immune cell function, a concept referred to as immunometabolism (42). T-regulatory cells, resting macrophages and naïve T-cells generate energy mostly through the Krebs cycle, whereas activated macrophages, B-cells, Th1, Th2 and Th17 cells shift the balance toward aerobic glycolysis to complement energy from Krebs cycle (43).

Thiamine is also involved with hemin-dependent oxygenase, whose action affects the release of the specific members of the intracellular adhesion molecule (ICAM) proteins. ICAMs bind integrins during immunological reactions, affecting T-cell activity and other immune system cells (16). Vitamin B1 is important in immune system reactivity as well because it plays a pivotal part in the expression of immunoglobulins and, due to its antioxidative effects on neutrophils, by protecting the sulfhydryl groups on the cell surfaces from oxidation (16, 42). On macrophages, thiamine suppresses the oxidative stress-induced activation of the necrosis factor NF-kB, which induces the release of a variety of inflammatory markers such as cytokines, growth factors and immune-responsive proteins (44).

Given the crucial role of thiamine in the maintenance of metabolic functions and its supposed effect on the pathophysiology of mitochondrial dysfunction and microcirculatory changes that are characteristic of critical illness, it is essential to better understand the effectiveness of thiamine supplementation in patients with septic shock.

## Single Thiamine Supplementation in Septic Shock

Several observational studies have explored the relationship between thiamine therapy and mortality rate in septic shock patients, with conflicting results (11, 14, 45–47). In a large retrospective matched cohort study, Woolum et al. (14) demonstrated that early intravenous (IV) thiamine supplementation (any dose between 100 and 500 mg) was associated with reduced 28-day mortality and improved lactate clearance. Additionally, lower mortality was also observed in septic shock patients with alcohol-use disorders who received thiamine within 48 h of sepsis onset (48).

In addition, a nationwide observational cohort study assessed the effect of thiamine administration on 28-day mortality in Japanese patients with septic shock over 7 years (46). The study included 68,571 patients, of which about 27% received 100 or 200 mg of IV thiamine within 2 days of admission. The findings of this large study did not support an association between early thiamine supplementation and a decrease in mortality. However, it is important to highlight the retrospective design of the study and the dose of thiamine used, which may be too low compared to the doses used in other studies (ranging from 200 to 500 mg daily).

To date, regardless of its relevance, only two randomized clinical trials have been published with IV thiamine supplementation as a single nutrient (13, 49). Donnino et al. (13) conducted the first randomized, double-blind, placebo-controlled trial. Patients with septic shock received 200 mg of IV thiamine twice daily or a matching placebo for 7 days or until hospital discharge. Contrary to expectations, supplementation was not effective in reducing serum lactate after the first 24 h of intervention. The prevalence of vitamin B1 deficiency among individuals was 35%, and supplementation was not associated with shock reversal time, disease severity or mortality. However, in the subset of patients with thiamine deficiency, lactate levels were lower after the first dose of supplementation (2.1 [1.4–2.5] mmol/L vs. 3.1 [1.9–8.3] mmol/L, *p* = 0.03), and a decrease in mortality was observed (2 [13%] vs. 6 [46%)], *p* = 0.047).

Despite this being a pioneering study, the results should be taken with caution. First, the effect of thiamine supplementation on lactate reduction and mortality was found in a small sample size (*n* = 28). Second, shock resuscitation treatment by itself would be able to reduce serum lactate. Third, some confounding variables such as illness severity and organ dysfunction scores among non-surviving patients who had baseline disabilities and did not receive supplementation are not known. Finally, lactate levels were assigned based on a predefined plan of patients who died before the 24-h period. Thus, an increase of 20%, 15% and 10% from baseline was imputed for patients who died before the 6-h, 6–12-h and 12–24-h point, respectively (6, 13).

Afterward, a secondary analysis of this trial was performed by Moskowitz et al. (50). The authors found significantly lower serum creatinine levels and a lower need for renal replacement therapy in the thiamine-treated group compared to the placebo group. As in the original trial (13) overall mortality was not different but raises the hypothesis of thiamine's role as a renal protective in septic shock.

More recently, another randomized clinical trial assessed the vasopressor-free days over 7 days in septic shock patients who received 200 mg of IV thiamine or placebo every 12 h for 7 days or until hospital discharge (49). On the one hand, thiamine supplementation was not associated with a reduction in the need for vasoactive drugs in the first week of ICU admission or with 28-day mortality. On the other hand, the study had interesting findings such as a reduction in vasopressor dependency index and in the serum lactate concentration 24 h after the initial supplementation. However, it is important to note that these findings are questionable due to the small sample size (*n* = 50) and the early stopping point, limiting the validity of the results. Table 1 describes the main studies that used single thiamine supplementation in patients with septic shock. Only two studies evaluated the concentration of vitamin B1 prior to its supplementation in patients with septic shock (13, 48). In addition, the studies involved patients with differences in sepsis severity, cause of infection, presence of risk factors and, when used, varied methods for assessing the vitamin concentration (11, 13–15, 46, 48–50). It is noteworthy that so far there are no reference values for detecting thiamine deficiency among septic patients. Therefore, it is essential to develop new studies that more assertively assess deficiency and establish specific reference values for septic patients to identify subgroups that can benefit more effectively from the treatment.

**Table 1:** Clinical studies evaluating single thiamine supplementation in septic shock.

**Authors**	**Diagnosis, number of patients and design**	**Dose and time**	**Results**
Donnino et al. (13). *Crit Care Med*	Septic shock n = 88 Randomized, double-blind clinical trial Primary outcome: lactate levels 24 hours after the first dose Secondary outcomes: time to shock reversal, severity of illness and mortality	Thiamine 200 mg IV or placebo twice daily for 7 days or until hospital discharge	35% of the patients were thiamine deficient There was no difference in lactate levels at 24 hours (median: 2.5 mmol/L [1.5 - 3.4] vs. 2.6 mmol/L [1.6 - 5.1], p = 0.40) Overall mortality was 43% There was no difference in the proportion of patients with shock reversal between the thiamine and placebo groups (74% vs. 71%, p = 0.81) and mortality was also similar in both groups (42% vs 44%, p=1.00) There was no difference in APACHE II score (p = 0.15) and SOFA score (p= 0.41) between the groups Among thiamine deficient patients, those in the thiamine group had statistically significantly lower lactate levels at 24 hours (median 2.1 mmol/L [1.4 - 2.5] vs. 3.1 [1.9 - 8.3], p = 0.03)
Moskowitz et al. (50). *Ann Am Thorac Soc*	Septic shock n = 70 Secondary analysis of a randomized, double-blind trial Primary outcome: requirement for renal replacement therapy	Thiamine 200 mg IV or placebo twice daily for 7 days or until hospital discharge	32.8% of patients were deficient in thiamine Mortality of 37.1% (32.2% in the thiamine group and 41% in the placebo group; p=0.45) Lower serum creatinine levels (p=0.05) and a lower need for renal replacement therapy in the thiamine-treated group compared to placebo (3% vs. 21%, p=0.04) No differences regarding APACHE II, SOFA score, time of MV and other clinicals and demographics variables between the groups.
Holmberg et al. (48). *J Crit Care*	Septic shock with alcohol use disorders n = 53 Retrospective Primary outcomes: Mortality and practice patterns relating to thiamine administration in patients with alcohol use disorders	Low-dose (100 mg) was the most frequently ordered dose. Median time to administration was 9 (4–18) h	Thiamine deficiency was not evaluated 64% of the patients received thiamine at hospital admission Thiamine administration was associated with decreased mortality (44% vs. 79%, p = 0.02) No differences regarding SOFA score, hospital, and ICU-free days and other clinicals and demographics variables between the groups
Woolum et al. (14). *Crit Care Med*	Septic shock n = 1,049 Retrospective Primary outcomes: lactate clearance Secondary outcomes: 28-days mortality, change in SOFA score, AKI or RRT within the ICU, vasopressor-free, ventilator-free, and ICU-free days within the 28 days following ICU admission	High-dose thiamine (500 mg) was the most frequently ordered dose. Thiamine was administered for a median of 3 days	Thiamine deficiency not evaluated The median time from hospital admission to thiamine administration was 6.4 hours Thiamine administration was associated with improved lactate clearance (hazard ratio, 1.307; 95% CI, 1.002–1.704) and a reduction in 28-day mortality (hazard ratio, 0.666; 95% CI, 0.490–0.905) There were no differences in any other secondary outcomes
Harun et al. (15). *Crit Care and Shock*	Septic shock n = 72 Randomized clinical trial Primary outcome: lactate clearance	Thiamine 200 mg IV or placebo twice daily for 3 days	Thiamine deficiency not evaluated Supplementation was not associated with relative lactate changes (p= 0.091) No differences regarding SOFA score, ICU LOS and ICU mortality
Miyamoto et al. (46). *Crit Care Med*	Septic shock n = 68,571 Retrospective Primary outcome: 28-day mortality	Low-dose (100 mg and 200 mg) were the most frequently ordered doses within 2 days of admission	Thiamine deficiency not evaluated No significant differences between the 100-mg thiamine group and the control group (risk difference, 0.6%; 95% CI, −0.3% to 1.4%) and the 200-mg thiamine group and the control group (risk difference, −0.3%; 95% CI, −1.3% to 0.8%) regarding mortality
Petsakul et al. (49). *BMC Anesthesiology*	Septic shock n = 50 Randomized clinical trial Primary outcome: decrease in vasopressor requirement within 7 days	Thiamine 200 mg IV or placebo twice daily for 7 days or until hospital discharge.	Thiamine deficiency was not evaluated No difference in vasopressor-free days between the thiamine and placebo groups (p = 0.197) There was a reduction in the dependence index on vasopressors (0.14 mmHg^−1^ vs.0.03 mmHg^−1^, p = 0.02) and in the serum lactate concentration at 24 h (1.0 mmol/L vs. 0.5 mmol/L, p = 0.024) after initial supplementation No difference was observed in SOFA score within 7 days, vasopressor dependency index within 4 days and 7 days, or 28-day mortality

*AKI, Acute kidney injury; APACHE II, Acute Physiology And Chronic Health Evaluation; IV, intravenous; ICU, intensive care unit; LOS, length of stay; MV, Mechanical ventilation; RRT, Renal replacement treatment; SOFA, sequential organ failure assessment*.

Studies with thiamine supplementation are still incipient, and therefore, it is essential to develop further clinical trials to conclusively determine the true role of thiamine in septic shock. Some problems related to the neutrality of these clinical trials are the study design, doses, route, timing, length of intervention and the choice of endpoints.

Recently, the concept that multi-micronutrient administration may be better than single-micronutrient administration has gained traction (12). In general, clinical trials consider the administration of a single micronutrient as a drug. However, the antioxidant defense is a complex system of endogenous agents in which micronutrients act as cofactors, and the physiological interactions between micronutrients are little discussed. Thus, current evidence does not support pharmacological use of single thiamine supplementation in septic shock patients, and future trials will probably focus on an early multi-micronutrient approach (12).

## Combined Thiamine Supplementation on the Prognosis of Patients With Septic Shock

The combined supplementation of thiamine with ascorbic acid and corticosteroids was tested in a retrospective before–after clinical study developed by Marik et al. (51). Patients with severe sepsis or septic shock were treated with the administration of the combination of vitamin C, (1.5 g every 6 h), hydrocortisone (50 mg every 6 h), and thiamine (200 mg every 12 h) for 4 days. During the control period, patients with sepsis did not receive intravenous vitamin C or thiamine. Surprisingly, the treated group had a significantly lower mortality rate than the control group (8.5 vs. 40.4%, *p* < 0.001). Despite this tremendous potential as a treatment for sepsis, this study warrants caution in extrapolating its results because notable limitations were found. In sum, the study had a small sample size (*n* = 47 patients), had one single-center and lacked blinding and randomization.

The use of low-dose corticosteroids is recommended in patients with septic shock who do not respond to vasopressors. Its main benefit is its immune-stimulating effects, which may limit the anti-inflammatory immunosuppressive state (52). Although mortality reduction is seen mainly in patients receiving higher vasopressor doses, its supplementation improves other secondary outcomes, such as shock recovery and ICU length of stay (53, 54). In addition, glucocorticoids and vitamin C appear to act synergistically in protecting or reversing endothelial dysfunction (55, 56) and have become an extremely interesting target in this population. Vitamin C deficiency is highly prevalent among critically ill patients and is related to increased need for vasopressors, larger organ dysfunction, kidney injury and shorter survival (54, 57). Despite its potent antioxidant action, the IV administration of vitamin C in high doses and for a long period requires caution due to the risk of hyperoxaluria and pro-oxidant action (55, 58). Additionally, the correction of thiamine deficiency acts as a cofactor in the oxidation of glyoxylate by the enzyme glyoxylate aminotransferase (56, 59) and may help to attenuate oxidative stress and inflammation in animal models of sepsis (55, 60). Consequently, a deficiency of both vitamins concomitantly can aggravate the oxidative mitochondrial injury and bioenergetic failure present in septic shock (54).

Despite the rationale for combining these vitamins with corticosteroids, the improvement in mortality was not reproducible in recent trials (55, 61–63). Subsequently, in search of more promising outcomes, studies were carried out with the supplementation of thiamine in higher doses associated with ascorbic acid and hydrocortisone. Table 2 summarizes the main prospective and randomized studies published to date on the effect of combination therapy with ascorbic acid, hydrocortisone and thiamine in patients with septic shock. These combined therapy studies were also discussed in some systematic reviews (64–67).

**Table 2:** Randomized clinical trials evaluating thiamine supplementation as adjunctive therapy in septic shock.

**Authors**	**Diagnosis, number of patients and design**	**Nutrients. dose and time**	**Results**	**Strong points/limitations**
VITAMINS Trial. Fugii et al. (61). *JAMA*	Septic shock n = 216 Multicentre, open-label, randomized clinical trial Primary outcomes: duration of time alive and free of vasopressor administration up to day 7 Secondary outcomes: 28-day, 90-day ICU, and hospital mortality, 28-day cumulative vasopressor-free days, 28-day cumulative mechanical ventilation-free days, 28-day renal replacement therapy–free days, change in SOFA score at day 3, 28-day ICU free-days, and hospital LOS	Intervention group: IV vitamin C (1.5 g every 6 h), hydrocortisone (50 mg every 6 h) and thiamine (200 mg every 12 h) Control group: IV hydrocortisone (50 mg every 6 h) Until shock resolution or up to 10 days	There was no significant difference in time alive and free of vasopressors up to day 7 (−0.6 h [95% CI, −8.3 to 7.2 h; p = 0.83]). There was no statistically significant difference in secondary outcomes.	Patients with septic shock within 24 h of diagnosis to maximize the possible effects of the intervention No serious adverse events were reported
HYVCTTSSS study. Chang et al. (62). *CHEST*	Sepsis and septic shock n = 80 Single-blind, randomized controlled trial Primary outcome: 28-day mortality. Secondary outcomes: duration of vasopressor use, ICU LOS, change in SOFA score within 72 h after experimental intervention, and PCT clearance rate within 72 h after experimental intervention	Intervention group: IV vitamin C (1.5 g every 6 h), hydrocortisone (50 mg every 6 h) and thiamine (200 mg every 12 h) Control group: placebo Hydrocortisone for 7 days and vitamin C and B1 for 4 days	There was no difference in mortality between the treatment and control groups (relative risk [RR],0.79; 95% CI, 0.41–1.52; p = 0.47) Thiamine treatment was associated with a significant improvement of 72-h change in ΔSOFA score (3.5 ± 3.3 vs. 1.8 ± 3.0; p = 0.02) and exhibited more incidents of hypernatremia (13 vs. 3; p = 0.005) In a subgroup diagnosed with sepsis within 48 h at ICU admission, an improvement in mortality in the treatment group was observed (13.6% vs 47.6%; RR, 0.29; 95% CI,0.09-0.90; p = 0.02)	Small sample size, single-blind and terminated early Did not include corticosteroids in the control group
ORANGES trial Iglesias et al. (55). *CHEST*	Sepsis and septic shock n = 137 Double-blind, randomized clinical trial Primary outcomes: resolution of shock and change in SOFA score Secondary outcomes: ICU mortality, hospital mortality, procalcitonin clearance (PCT-c), LOS, ICU LOS, and ventilator-free day	Intervention group: IV vitamin C (1.5 g every 6 h), hydrocortisone (50 mg every 6 h) and thiamine (200 mg every 12 h) Control group: placebo Maximum of 4 days	No statistically significant change in SOFA score was found between groups (p = 0.17) Intervention group showed quicker reversal of shock (27 ± 22 h vs 53 ± 38 h; p < 0.001) No significant differences were found between study mortality, length of stay and ventilator-free days	Baseline ascorbic acid and thiamine levels were evaluated Homogenous (primarily white) cohort size, limiting the ability to detect differences in hospital mortality and length of stay Did not include corticosteroids in the control group
Wani et al. (63). Infectious Disease	Sepsis and septic shock n = 100 Open-label, randomized controlled trial Primary outcomes: hospital mortality Secondary outcomes: 30-day mortality, duration of hospital stay, duration of vasopressor therapy, lactate clearance, change in serum lactate and the SOFA score over the first 4 days	Intervention group: IV vitamin C (1.5 g every 6 h), hydrocortisone (50 mg every 6 h) and thiamine (200 mg every 12 h) Control group: placebo Vitamin C and B1 for 4 days or until discharge from hospital; hydrocortisone for 7 days or until discharge from hospital	There was no difference between groups regarding hospital mortality (p = 0.82) and 30-day mortality (p = 1.00) Intervention group had shorter vasopressor use (96.13 ± 40.50 h vs. 75.72 ± 30.29 h; p = 0.010) and greater lactate clearance compared to control (41.8% vs. 56.8%; p = 0.031) No difference in mortality, length of stay and SOFA score	Geographical area (India) with high prevalence of antimicrobial resistance and mortality from sepsis Open-label and small sample size Did not include corticosteroids in the control group
ACTS trial Moskowitz et al. (38). *JAMA*	Septic shock n = 200 Multicentre, randomized, blinded clinical trial Primary outcome: change in the SOFA score between enrolment and 72-hour follow-up Secondary outcomes: kidney failure, 30-day mortality, ventilator-free days, and shock-free days during the first 7 days, days free of ICU stay, all-cause mortality to ICU and hospital discharge, post hospitalization disposition in survivors to hospital discharge, 72-hour change in individual SOFA score components, and delirium on day 3	Intervention group: IV vitamin C (1.5 g every 6 h), hydrocortisone (50 mg every 6 h) and thiamine (100 mg every 6 h) Control group: placebo For 4 days or until discharge from ICU	There was no statistically significant difference in SOFA score between groups (p = 0.12) The median number of shock-free days was higher in the intervention group compared with the placebo group (5 [IQR, 3–5] days vs 4 [IQR, 1–5] days; median difference, 1.0 days; 95% CI, 0.2-1.8 days; p < 0.01) There was no statistically significant difference in any other secondary outcomes	Conducted at 14 centres Adverse events were hyperglycaemia, hypernatremia and new hospital-acquired infection Large number of patients were screened (n = 4,569) but not randomized Did not include corticosteroids in the control group
ATESS trial. Hwang et al. (68). *Intensive Care Medicine*	Septic shock n = 111 Multicentre, double-blind, randomized clinical trial Primary outcomes: ΔSOFA score Secondary outcomes: 7-day, 28-day, 90-day, in-hospital and ICU mortality, shock reversal, vasopressor free days, vasopressor dose, duration of mechanical ventilation, ventilator-free days, AKI, RRT, RRT-free days, LOS ICU, ICU-free days, hospital LOS, reduction of C-reactive protein (CRP) and procalcitonin for 72 h	Intervention group: IV vitamin C (50 mg/kg, every 12 h, maximum daily dose 6 g) and thiamine (200 mg every 12 h) Control group: placebo For 48 h	There was no significant difference in ΔSOFA scores between the treatment group and the placebo group (3, interquartile range IQR – 1 to 5 vs. 3, IQR 0–4, respectively, p=0.96]) There was no significant difference in any secondary outcomes.	Glucocorticoid was administered to over half of the patients Interval for vitamin administration was longer (12 h vs. 6 h), while the duration of treatment was shorter (48 h vs. 96 h or more) compared to previous studies Intra-abdominal infection, either solid cancer or hematologic malignancy, accounted for almost half of the cases of septic shock
VICTAS Randomized Clinical Trial. Sevransky et al. (69). *JAMA*	Sepsis n = 501 Multicentre, double-blind, randomized clinical trial Primary outcomes: ventilator- and vasopressor-free days in the first 30 days Secondary outcomes: 30-day mortality	Intervention group: IV vitamin C (1.5 g), thiamine (100 mg), and hydrocortisone (50 mg) every 6 h Control group: hydrocortisone (of at least 200 mg) or matching placebo equivalent For 96 h or until discharge or death	There was no statistically significant difference between the intervention and control groups regarding ventilator- and vasopressor-free days (median difference of −1 day [95% CI, −4 to 2 days; p = 0.85]) There was no difference between groups in 30-day mortality (intervention = 22% vs placebo = 24%, p = 0.619)	Trial was terminated early for administrative reasons and may have been underpowered to detect a clinically important difference

*AKI, Acute kidney injury; CI, confidence interval; CRP, C-reactive protein; IV, intravenous; ICU, intensive care unit; LOS, length of stay; PCT-c, Procalcitonin clrearence; RR, razard ratio; IQR, interquartile range; RRT, Renal replacement treatment; SOFA, sequential organ failure assessment*.

To date, fewer than 10 randomized clinical studies have been published with combined thiamine supplementation in patients with sepsis and/or septic shock (38, 55, 61–63, 68, 69). The main outcomes involved organ dysfunction, time of need for vasopressor, ventilator-free days, development of acute kidney injury, lactate clearance, length of stay in the ICU and hospital mortality. Most studies did not observe any statistically significant difference regarding outcomes between treated individuals and the control group. Only in the study of Wani et al. (63) did the intervention group have shorter vasopressor use (96.13 ± 40.50 h vs. 75.72 ± 30.29 h, *p* = 0.010) and greater lactate clearance (41.81 vs. 56.83%, *p* = 0.031) compared to the control. It is noteworthy that the study in question has limitations, including being open-label, having a small sample size and having a high prevalence of antimicrobial resistance and mortality.

If the lack of beneficial effects of supplementation on the outcomes is evident, the assertiveness in the design of the work brings some questions. Between studies, doses are generally similar (vitamin C: 1,500 mg; hydrocortisone: 50 mg; thiamine: 100 mg, every 6 h); however, the administration time is highly variable (48 h, 4–10 days or until discharged from the ICU). Even with a more diverse design, the ATESS trial (68) did not include the administration of hydrocortisone in the treated group, and the interval for vitamin administration was longer (12 vs. 6 h), while the duration of treatment was shorter (48 vs. 96 h or more) compared to previous studies.

It is important to note that hydrocortisone monotherapy in patients with septic shock is associated with faster resolution of shock (70) and lower mortality (71) when compared with placebo groups. In this sense, the VITAMINS trial (61) compared IV thiamine combined with vitamin C and hydrocortisone with hydrocortisone administration alone and found no improvement on mortality or time free from vasopressors up to day 7, suggesting no synergic effect between them as previously postulated.

Still, for clinical practice, some questions still need to be clarified about the combined therapy for adjuvant management of septic shock. The current literature cannot sufficiently support use of single thiamine or combined administration outside of randomized controlled trials because most of the studies were interrupted early (69), a large number of patients were screened but not randomized (38), abdominal infections and tumors were highly prevalent (68) and the studies had mixed designs.

However, these efforts have unquestionably advanced care of septic shock patients. At the moment, it has been established that single thiamine administration or thiamine administration combined with vitamin C and hydrocortisone has a good safety profile with no adverse events, even at high doses. Consequently, studies are needed to fill the knowledge gaps regarding vitamin B1 supplementation as adjuvant therapy in septic shock.

## Conclusion

Studies with thiamine supplementation in septic shock have notable differences in design, including dosage and time of supplementation; small sample sizes; and different septic phenotypes. Despite the excellent safety profile, good biologic rationale and promising clinical studies, no robust results support routine thiamine supplementation to improve outcomes. However, future trials should focus on combined multi-micronutrient therapy, higher doses, and early administration, which might be the key to improving mitochondrial function and reducing oxidative stress during hemodynamic resuscitation in patients with septic shock.

## Author Contributions

NC, AP, and LG: conceptualization, methodology, and writing—original draft. CS and NV: revision of the original draft. PA, SP, and LZ: writing—review and editing. BP and MM: writing—original draft and writing—review and editing and supervision. All authors revised the article critically for important intellectual content and approved the final version of the manuscript.

## Conflict of Interest

The authors declare that the research was conducted in the absence of any commercial or financial relationships that could be construed as a potential conflict of interest.

## Publisher's Note

All claims expressed in this article are solely those of the authors and do not necessarily represent those of their affiliated organizations, or those of the publisher, the editors and the reviewers. Any product that may be evaluated in this article, or claim that may be made by its manufacturer, is not guaranteed or endorsed by the publisher.
